# Energy, Exergy, Exergoeconomic and Exergoenvironmental Impact Analyses and Optimization of Various Geothermal Power Cycle Configurations

**DOI:** 10.3390/e23111483

**Published:** 2021-11-09

**Authors:** Moein Shamoushaki, Mehdi Aliehyaei, Marc A. Rosen

**Affiliations:** 1 Department of Industrial Engineering, University of Florence, 50134 Firenze, Italy; moein.shamoushaki@unifi.it; 2 Department of Mechanical Engineering, Pardis Branch, Islamic Azad University, Pardis New City 1468995513, Iran; 3 Faculty of Engineering and Applied Science, University of Ontario Institute of Technology, 2000 Simcoe Street North, Oshawa, ON L1G 0C5, Canada; marc.rosen@uoit.ca

**Keywords:** geothermal, ORC, exergy, exergoeconomic, optimization

## Abstract

Energy, exergy, and exergoeconomic evaluations of various geothermal configurations are reported. The main operational and economic parameters of the cycles are evaluated and compared. Multi-objective optimization of the cycles is conducted using the artificial bee colony algorithm. A sensitivity assessment is carried out on the effect of production well temperature variation on system performance from energy and economic perspectives. The results show that the flash-binary cycle has the highest thermal and exergy efficiencies, at 15.6% and 64.3%, respectively. The highest generated power cost and pay-back period are attributable to the simple organic Rankine cycle (ORC). Raising the well-temperature can increase the exergy destruction rate in all configurations. However, the electricity cost and pay-back period decrease. Based on the results, in all cases, the exergoenvironmental impact improvement factor decreases, and the temperature rises. The exergy destruction ratio and efficiency of all components for each configuration are calculated and compared. It is found that, at the optimum state, the exergy efficiencies of the simple organic Rankine cycle, single flash, double flash, and flash-binary cycles respectively are 14.7%, 14.4%, 12.6%, and 14.1% higher than their relevant base cases, while the pay-back periods are 10.6%, 1.5% 1.4%, and 0.6% lower than the base cases.

## 1. Introduction

In recent decades, increases in world population and energy demand have led to increases in fossil fuel consumption, leading to greenhouse gas emissions and related global warming and climate change, as well as stratospheric ozone layer destruction. The use of renewable energies and modern technologies with lower environmental impacts is an option to address these problems. Geothermal energy is a promising renewable energy for generating electricity from low-temperature heat sources. Geothermal energy includes several advantageous features, such as reliability of supply and sustainability, with a temperature range varying from 50 to 350 °C [1,2,3].

Coskun et al. [4] proposed a modified exergoeconomic approach using exergy and cost-accounting analyses for geothermal power plants. They evaluated the relationship between capital costs and exergy loss/destruction for the system components. Yildirim and Ozgener [5] examined two geothermal power plants (DORA I and DORA II) with thermodynamic and exergoeconomic concepts. The effect of working fluid on energy and exergy efficiencies was assessed, and the main sections that destroy exergy were determined. Kecebas [6] investigated geothermal district heating systems from energy, exergy, economic, and environmental viewpoints. He applied actual operation data to evaluate the system performance, including first and second law efficiencies, specific exergy index, exergetic improvement potential, and exergy losses. Al-Ali and Dincer [7] carried out energy and exergy analyses of a multigenerational integrated geothermal and solar system used to produce electricity, cooling, heating, and hot water. They found that the exergy efficiency of the multi-generation system was 36.6%, and that of the single generation was 26.2%.

Zare [8] assessed and compared three organic Rankine cycle (ORC) configurations for binary geothermal power plants from thermodynamic and economic viewpoints, finding that an ORC with an internal heat exchanger has better thermodynamic performance than other configurations. Yari et al. [9] carried out thermodynamic and exergoeconomic evaluations of a trilateral power cycle, and compared the results with those for ORC and Kalina cycles. Their results showed that the trilateral Rankine cycle net power is higher than that for the other considered cycles. Shokati et al. [10] analyzed and optimized various geothermal configurations, such as basic, dual-pressure, dual-fluid ORC, and Kalina cycles, from exergy and exergoeconomic points of view. They showed that the turbine in the basic and Kalina cycles, and the low-pressure turbine in the dual-pressure and dual-fluid ORCs, have the highest total cost rates. Zhao and Wang [11] performed an exergoeconomic analysis of a flash-binary geothermal cycle and its optimization, as well as a parametric evaluation of impact on system performance of varying such parameters, as flash pressure, ORC turbine inlet pressure, and ORC turbine inlet temperature.

Calise et al. [12] performed exergetic and exergoeconomic assessments of a hybrid renewable system that includes geothermal and solar energies as heat resources. Their results showed that the exergy efficiency varies between 40% to 50% in the winter, and 16% to 20% in the summer. Shamoushaki et al. [13] carried out energy, exergy, exergoeconomic, and environmental assessments of a solid oxide fuel cell and gas turbine (SOFC-GT) system. They carried out multi-objective optimization of the considered system with the non-dominated sorting genetic *algorithm II* (NSGA-II). Fiaschi et al. [14] performed exergy and exergoeconomic analyses of low and medium temperature geothermal plants in Italy. They showed that the system with R1233zd (E) as the working fluid has the best exergoeconomic performance among the considered cycles. Bianchi et al. [15] studied a micro-ORC case study using geothermal energy, and showed that the expander and feed pump efficiencies and the ORC efficiency at the geothermal well-working conditions were 53%, 41%, and 4.4%, respectively. Behzadi et al. [16] conducted thermodynamic and exergoeconomic assessments of an integrated energy system with geothermal and solar heat resources. They performed a multi-objective optimization of this system, considering exergy efficiency and total product unit cost as objective functions.

Ozkaraca [17] examined a binary geothermal power cycle from an advanced exergy perspective. They also optimized the system using artificial bee colony methods. Exergy efficiencies from the conventional exergy assessment, the advanced exergy evaluation, and the optimization algorithm were found to be 39.1%, 43.1%, and 42.8%, respectively. Manente et al. [18] analyzed and compared integrated flash-binary and two-phase binary plants. The two considered plant layouts integrate water absorption and reinjection of H_2_S and CO_2_. Ehyaei et al. [19] carried out exergy and exergoeconomic analyses of a combined geothermal cooling and power system. Their considered system was an ORC as an upstream cycle, and an absorption chiller as a downstream cycle. Samadi and Kazemi [20] studied a new combined ORC and geothermal cycle with a zeotropic working fluid exergoeconomically. They also optimized the operating parameters of the cycle, and found that increasing the isobutane mole fraction in the isobutane/isopentane mixture causes a reduction in economic return on investment. Wang et al. [21] conducted thermodynamic and exergy analyses of a power and cooling generation system based on geothermal flash and organic Rankine cycles, and an ejector refrigeration cycle. They applied various working fluids, and showed that a system with isopentane (0.3)/R142b (0.7) as a working fluid had the highest exergy efficiency and lowest exergy destruction.

Abdolalipouradl et al. [22] evaluated four flash geothermal power cycles from thermodynamic and exergoeconomic viewpoints. They carried out comprehensive parametric studies for the configurations, considering several ORC working fluids. Shamoushaki et al. [23] performed exergy, exergoeconomic, and exergoenvironmental analysis of a flash-binary geothermal power cycle to generate electricity, heating, and cooling. The optimization results showed that the total cost rate at the optimum state is 10.3% lower and the exergetic efficiency is 4.5% higher than the base case. Ding et al. [24] conducted an exergoeconomic evaluation of a flash-binary geothermal plant to generate power and cooling. They applied a zeotropic fluid in their assessment in the system’s binary part. The optimization of the system was performed with the NSGA-II algorithm. Ehyaei et al. [3] proposed a novel geothermal system that included a combined cooling and power system with reverse osmosis and sodium hypochlorite. The energy efficiency, exergy efficiency, and pay-back period were shown to be 12.25%, 19.6%, and 2.7 years, respectively.

In this study, four geothermal power cycles are evaluated from the perspectives of energy, exergy, exergoeconomic, and exergoenvironmental impact. The thermodynamic modeling is performed with MATLAB using Refprop 9.1 [25]. Simple organic Rankine, single flash, double flash, and flash-binary cycles are investigated. The applied working fluid in the ORC system is R245fa. The main objective of this study is to compare the operational performances of various geothermal configurations from different perspectives in order to consider their critical points. An additional objective of the present research is to determine the best operational parameters values in all cases by optimizing them. A sensitivity analysis is performed of the impact of production well temperature on system performance. The main thermodynamic, economic, and environmental parameters are compared for the cycles. Multi-objective optimization of the configurations is carried out. Optimization is performed using the artificial bee colony algorithm, which is a powerful method to optimize numerical problems. The design variables and main operational parameters for optimum states and base cases are compared for all configurations. The novelty of this work is as follows:

- Studying four different geothermal configurations from exergy, exergoeconomic, and exergoenvironmental impact viewpoints;
- Comparing the main economic parameters of these system configurations, including levelized cost of electricity, pay-back period, and electricity cost;
- Comparing the exergoenvironmental impacts of the configurations through exergoenvironmental impact improvement and exergetic sustainability indexes;
- Optimization of all configurations by the artificial bee colony algorithm to find the best operation and modify systems based on the optimization.

## 2. Energy and Exergy Modeling

**Figure 1:**
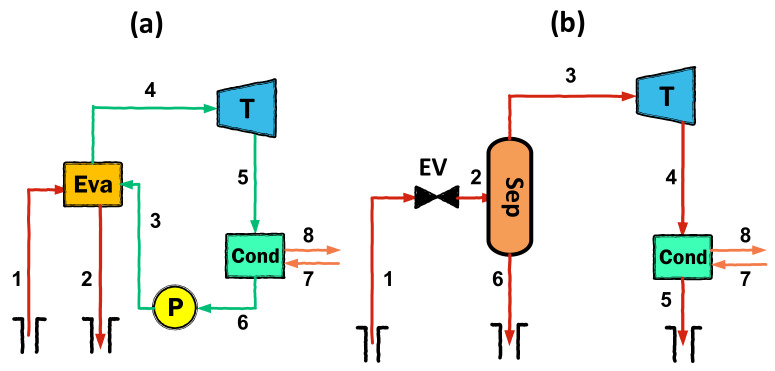
Schematic diagram of (**a**) simple ORC and (**b**) single flash cycle.

**Figure 2:**
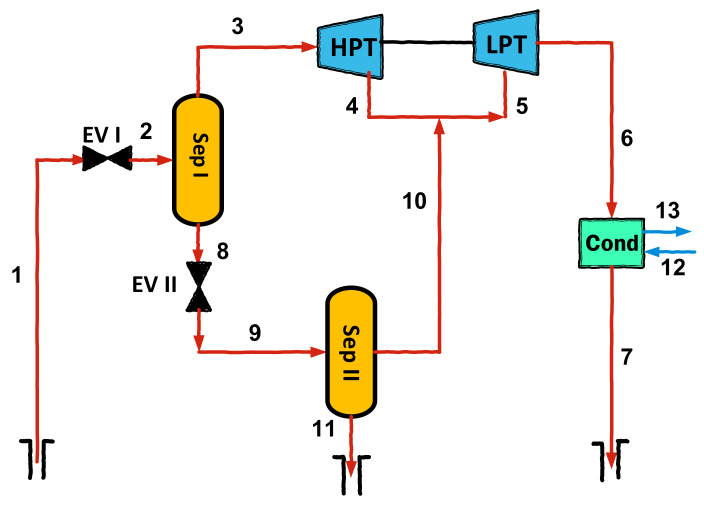
Schematic diagram of double flash cycle.

**Figure 3:**
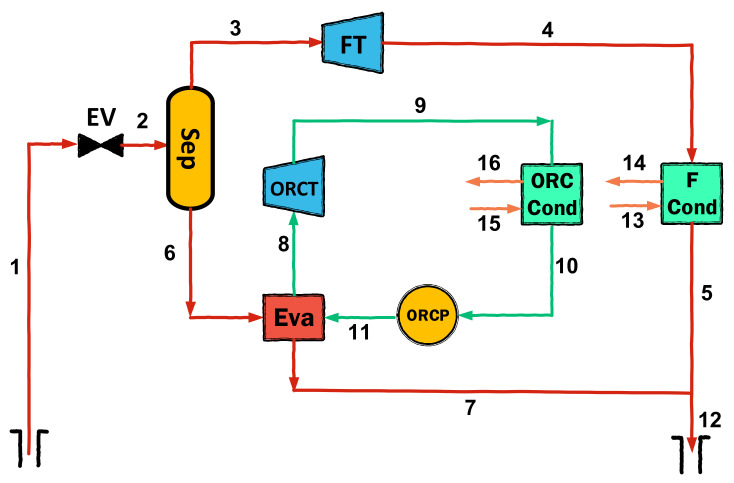
Schematic diagram of double flash-binary cycle.

Mathematical modeling is carried out in accordance with the first and second laws of thermodynamics, using mass, energy, and exergy balance equations. Diagrams of the considered geothermal configurations are given in Figure 1, Figure 2 and Figure 3. The assumptions used in the thermodynamic modeling and analysis are as follows:

- All cycles operate under steady-state conditions [26];
- Potential and kinetic energies are negligible [19];
- The ambient temperature and pressure are fixed at 298.15 K and 1.013 kPa, respectively [27];
- The pump and turbines have fixed isentropic efficiencies [19];
- Pressure losses in pipes and all components are negligible [28].

General mass and energy rate balance equations follow:

(1) $$\sum {\dot{m}}_{i}=\sum {\dot{m}}_{e}$$

(2) $$\sum {\left(\dot{m}h\right)}_{i}+\dot{Q}=\sum {\left(\dot{m}h\right)}_{e}+\dot{W}$$

Here, subscripts i and e represent the control volume inlet and outlet respectively, while $\dot{m}$, h, $\dot{Q},$ and $\dot{W}$ are mass flow rate $\left(\mathrm{kg}/s\right)$, specific enthalpy $\left(\mathrm{kJ}/\mathrm{kg}\right)$, heat transfer rate, and work rate respectively $\left(\mathrm{kW}\right)$. Operational parameters applied for modeling the systems are presented in Table 1. A general exergy rate balance equation can be written as follows [29,30]:

(3) $$\sum {\left(\dot{m}.ex\right)}_{i}+\dot{E}x_{q}=\sum {\left(\dot{m}.ex\right)}_{e}+\dot{E}x_{w}+\dot{E}x_{D}+\dot{E}x_{L}$$

Here, ex denotes specific exergy of a stream $\left(\mathrm{kJ}/\mathrm{kg}\right)$, while $\dot{E}x_{Q}$, $\dot{E}x_{W}$, $\dot{E}x_{D}$, and $\dot{E}x_{L}$ are the exergy rates of heat transfer, work, exergy destruction, and loss for a component $\left(\mathrm{kW}\right)$, respectively. Exergy can be divided into four parts: kinetic, potential, physical, and chemical. In this study, chemical, kinetic, and potential parts of exergy are considered insignificant. The physical exergy of stream k can be written as [29,31]:

(4) $$ex_{ph,k}={\left(h-h_{0}\right)}_{k}-T_{0}{\left(s-s_{0}\right)}_{k}$$

where 0 refers to the reference environment condition, which is taken to be the same as the ambient condition. Moreover, s is specific entropy $\left(\mathrm{kJ}/\mathrm{kg}.K\right)$, and subscript k refers to the kth stream. Exergy destruction and loss equations for the devices in the considered cycles are presented in Table 2. In the exergy analysis of a system, one of the most significant variables is the exergy destruction ratio, which is defined as the exergy destruction rate of a device divide into total exergy destruction rate [14]:

(5) $$y_{D,k}=\frac{\dot{E}x_{D_{k}}}{\dot{E}x_{D_{Tot}}}$$

**Table 1:** Cycle input parameters for thermodynamic modeling.

Cycle	Parameter	Unit	Value	Reference
Simple ORC	$P_{1}$	kPa	2797	[32]
	$T_{1}$	K	503	[32]
	${\dot{m}}_{1}$	kg/s	100	-
	$P_{4}$	kPa	3000	-
	$T_{2}$	K	313	-
	$T_{6}$	K	303	-
	$x_{6}$	-	0	-
	$\eta_{p}$	%	85	[19]
	$\eta_{T}$	%	85	[19]
Single flash	$P_{1}$	kPa	2797	[32]
	$T_{1}$	K	503	[32]
	${\dot{m}}_{1}$	kg/s	100	-
	$P_{2}$	kPa	600	-
	$T_{5}$	K	313	-
	$\eta_{FT}$	%	85	[19]
Double flash	$P_{1}$	kPa	2797	[32]
	$T_{1}$	K	503	[32]
	${\dot{m}}_{1}$	kg/s	100	-
	$P_{2}$	kPa	600	-
	$P_{9}$	kPa	200	-
	$T_{7}$	K	313	-
	$\eta_{HPT}$	%	85	[19]
	$\eta_{LPT}$	%	85	[19]
Flash-binary	$P_{1}$	kPa	2797	[32]
	$T_{1}$	K	503	[32]
	${\dot{m}}_{1}$	kg/s	100	-
	$P_{2}$	kPa	600	-
	$P_{8}$	kPa	3000	-
	$T_{10}$	K	303	-
	$x_{10}$	-	0	-
	$T_{12}$	K	313	-
	$\eta_{ORCP}$	%	85	[19]
	$\eta_{FT}$	%	85	[19]
	$\eta_{ORCT}$	%	85	[19]

Moreover, the exergy efficiency for device *k* can be written as:

(6) $$\eta_{ex,k}=\frac{\dot{E}x_{P,k}}{\dot{E}x_{F,k}}$$

Here, $\dot{E}x_{P,k}$ and $\dot{E}x_{F,k}$ is the exergy flow rate of product(s) and fuel(s), respectively. Based on the SPECO approach, some components destroy exergy and produce nothing, such as valves and condensers, for which exergy loss should be considered. Thermal (or energy), and exergy efficiencies of a system configuration can be expressed as:

(7) $$\eta_{th}=\frac{{\dot{W}}_{net}}{{\dot{Q}}_{in}}$$

(8) $$\eta_{ex}=\frac{{\dot{W}}_{net}}{\dot{E}x_{i}}$$

The ${\dot{Q}}_{in}$ and $\dot{E}x_{i}$ for all systems are intriduced as based on energy/exergy input from production wells.

**Table 2:** Exergy destruction rate equations for components in the cycles.

Cycle	Component	Exergy Destruction	Exergy Loss
Simple ORC	P	$\dot{E}x_{D,P}=\dot{E}x_{6}-\dot{E}x_{3}+{\dot{W}}_{P}$	-
	Eva	$\dot{E}x_{D,Eva}=\dot{E}x_{1}+\dot{E}x_{3}-\dot{E}x_{2}-\dot{E}x_{4}$	-
	T	$\dot{E}x_{D,T}=\dot{E}x_{4}-\dot{E}x_{5}-{\dot{W}}_{T}$	-
	Cond	$\dot{E}x_{D,Cond}=\dot{E}x_{5}+\dot{E}x_{7}-\dot{E}x_{6}-\dot{E}x_{8}$	$\dot{E}x_{L,Cond}=\dot{E}x_{8}-\dot{E}x_{7}$
Single flash	EV	$\dot{E}x_{D,EV}=\dot{E}x_{1}-\dot{E}x_{2}$	-
	Sep	$\dot{E}xD_{Sep}=\dot{E}x_{2}-\dot{E}x_{3}-\dot{E}x_{6}$	-
	T	$\dot{E}x_{D,T}=\dot{E}x_{3}-\dot{E}x_{4}-{\dot{W}}_{T}$	-
	Cond	$\dot{E}x_{D,Cond}=\dot{E}x_{4}+\dot{E}x_{7}-\dot{E}x_{5}-\dot{E}x_{8}$	$\dot{E}x_{L,Cond}=\dot{E}x_{8}-\dot{E}x_{7}$
Double flash	EV I	$\dot{E}x_{D,EV,I}=\dot{E}x_{1}-\dot{E}x_{2}$	-
	Sep I	$\dot{E}x_{D,Sep,I}=\dot{E}x_{2}-\dot{E}x_{3}-\dot{E}x_{8}$	-
	EV II	$\dot{E}x_{D,EV,II}=\dot{E}x_{8}-\dot{E}x_{9}$	-
	Sep II	$\dot{E}x_{D,Sep,HH}=\dot{E}x_{9}-\dot{E}x_{10}-\dot{E}x_{11}$	-
	HPT	$\dot{E}x_{D,HPT}=\dot{E}x_{3}-\dot{E}x_{4}-{\dot{W}}_{HPT}$	-
	LPT	$\dot{E}x_{D,LPT}=\dot{E}x_{5}-\dot{E}x_{6}-{\dot{W}}_{LPT}$	-
	Cond	$\dot{E}x_{D,Cond}=\dot{E}x_{6}+\dot{E}x_{12}-\dot{E}x_{7}-\dot{E}x_{13}$	$\dot{E}x_{L,Cond}=\dot{E}x_{13}-\dot{E}x_{12}$
Flash-Binary	EV	$\dot{E}x_{D,EV}=\dot{E}x_{1}-\dot{E}x_{2}$	-
	Sep	$\dot{E}x_{D,Sep}=\dot{E}x_{2}-\dot{E}x_{3}-\dot{E}x_{6}$	-
	FT	$\dot{E}x_{D,FT}=\dot{E}x_{3}-\dot{E}x_{4}-{\dot{W}}_{FT}$	-
	FCond	$\dot{E}x_{D,FCond}=\dot{E}x_{4}+\dot{E}x_{13}-\dot{E}x_{5}-\dot{E}x_{14}$	$\dot{E}x_{L,FCond}=\dot{E}x_{14}-\dot{E}x_{13}$
	Eva	$\dot{E}x_{D,Eva}=\dot{E}x_{6}+\dot{E}x_{11}-\dot{E}x_{7}-\dot{E}x_{8}$	-
	ORCT	$\dot{E}x_{D,ORCT}=\dot{E}x_{8}-\dot{E}x_{9}-{\dot{W}}_{ORCT}$	-
	ORC Cond	$\dot{E}x_{D,ORCCond}=\dot{E}x_{4}+\dot{E}x_{15}-\dot{E}x_{5}-\dot{E}x_{16}$	$\dot{E}x_{L,ORCCond}=\dot{E}x_{16}-\dot{E}x_{15}$
	ORC P	$\dot{E}x_{D,ORCP}=\dot{E}x_{11}-\dot{E}x_{10}+{\dot{W}}_{ORCP}$	-

## 3. Exergoeconomic Modeling

Exergoeconomics is a powerful and influential technique, driven by a combination of economic and exergy concepts, that helps researchers better understand systems from energy and economic viewpoints. Exergoeconomics permits economic designs of energy systems that are not obtainable by common economic modeling [29]. In the present study, the specific exergy costing (SPECO) method is applied in the exergoeconomic evaluation [33]. For the exergoeconomic modeling of each system, cost-rate balances and auxiliary equations are applied. Cost-rate balance equations for device *k* can be written as [29,34]:

(9) $${\dot{C}}_{q,k}+\sum {\dot{C}}_{i,k}+{\dot{Z}}_{k}={\dot{C}}_{w,k}+\sum {\dot{C}}_{e,k}$$

Here, ${\dot{C}}_{q,k}$ is unit cost rate of heat transfer $\left(\$/s\right)$, ${\dot{C}}_{w,k}$ is unit cost rate of work $\left(\$/s\right)$, and ${\dot{Z}}_{k}$ is capital cost rate. Moreover, ${\dot{C}}_{i,k}$ and ${\dot{C}}_{e,k}$ are the inlet and outlet cost units $\left(\$/s\right)$, respectively. Unit cost rate can be written as [29,34]:

(10) $${\dot{C}}_{k}=c_{k}\dot{E}x_{k}$$

In the exergoeconomic analysis of each system, there is dissipative equipment such as the condenser, valves, and so on, which are components without product in terms of total exergy. In general, for productive devices, any exit and inlet flow materials are product and resource. The product of dissipative components cannot be expressed in terms of total exergy. For a dissipative component, such a condenser, the cost balance should be defined as follows [33]:

(11) $${\dot{C}}_{e,k}+{\dot{C}}_{diff}={\dot{C}}_{i,k}+{\dot{Z}}_{k}$$

The component in this equation is a fictitious cost-rate-associated dissipative device. For the condenser as dissipative equipment, ${\dot{C}}_{diff}$ derived could be distributed amongst all other components, by using the entropy increment in each device as a weighting factor [33]. This approach is applied based on the dissipative element of all configurations.

$c_{k}$ and $\dot{E}x_{k}$ are cost per unit of energy $\left(\$/\mathrm{kJ}\right)$ and exergy rate of the *k*th stream of the cycle $\left(\mathrm{kW}\right)$, respectively. The total cost rate of the cycle is the sum of capital investment (CI) and operating and maintenance (O&M) costs [29]:

(12) $${\dot{Z}}_{k}={\dot{Z}}_{k}^{CI}+{\dot{Z}}_{k}^{OM}=\frac{Z_{k}\times CRF\times \varphi }{N\times 3600}$$

Here, $Z_{k}$, φ and N denote respectively the investment cost of the *k*th component $\left(\$\right)$, maintenance factor, and the annual plant working hours (which is considered 7446 h [35]). The purchasing cost correlations are listed in Table 3 respectively. The capital recovery factor $\left(CRF\right)$ can be expressed as [29,36]:

(13) $$CRF=\frac{i{\left(1+i\right)}^{n}}{{\left(1+i\right)}^{n}-1}$$

Here, i is the interest rate, which is considered 3%, and n is the lifetime of the power plant, which is assumed to be 30 years. In the exergoeconomic analysis, by introducing for each component product and fuel, the product and fuel cost of the components can be calculated. Moreover, the cost rate related to exergy destruction can be obtained by multiplying specific fuel cost and exergy destruction rate of each device [29]. Thus:

(14) $${\dot{C}}_{P,k}=c_{P,k}\dot{E}x_{P,k}$$

(15) $${\dot{C}}_{F,k}=c_{F,k}\dot{E}x_{F,k}$$

(16) $${\dot{C}}_{D,k}=c_{F,k}\dot{E}x_{D,k}$$

Here, $c_{P,k}$ and $c_{F,k}$ are the specific costs of product and fuel $\left(\$/\mathrm{kJ}\right)$, respectively, and ${\dot{C}}_{D,k}$ is exergy destruction cost rate of the *k*th component $\left(\$/s\right)$. The purchased equipment costs are given in Table 3. The constant coefficient of the Turton purchasing cost correlation can be found in [37]. Furthermore, the exergoeconomic factor and relative cost difference can expressed respectively as follows [29,38,39]:

(17) $$f_{k}=\frac{{\dot{Z}}_{k}}{{\dot{Z}}_{k}+{\dot{C}}_{D,k}+{\dot{C}}_{L,k}}$$

(18) $$r_{k}=\frac{c_{P,k}-c_{F,k}}{c_{F,k}}$$

The CEPCI (Chemical Engineering Plant Cost Index) is applied to consider annual inflation. This index is updated to the year 2020 [40,41]:

(19) $${PEC}_{k}=Z_{k}\times \left(\frac{{CEPCI}_{2020}}{{CEPCI}_{2001}}\right)$$

The geothermal well drilling cost for Iran can be determined from [41]:

(20) $$Well\;drilling\;cost=5.209\times 10^{5}n.log\left(z\right)+0.1982\times n.d^{2}+8.594\times 10^{5}$$

**Table 3:** Capital cost expressions for components of the system.

Component	Cost Correlation	Reference
Pump	$Z_{P}=1120\times {\left({\dot{W}}_{P}\right)}^{0.8}$	[42]
Turbine	$Z_{T}=6000\times {\left({\dot{W}}_{T}\right)}^{0.7}$	[43]
Condenser	$\log C_{0,Cond}=\left[K_{1,Cond}+K_{2,Cond}\left(\log A_{Cond}\right)+K_{3,Cond}{\left(\log A_{Cond}\right)}^{2}\right]$ $\log F_{P,Cond}=\left[C_{1,Cond}+C_{2,Cond}\left(\log P_{Cond}\right)+C_{3,HX}{\left(\log P_{Cond}\right)}^{2}\right]$ $Z_{Cond}=C_{0,Cond}\times \left[B_{1,Cond}+\left(B_{2,Cond}\times F_{M,Cond}\times F_{P,Cond}\right)\right]$	[37]
Expansion valve	$Z_{EV}=114.5\times \dot{m}$	[44]
Separator	$Z_{Sep}=280.3\times {\left(\dot{m}\right)}^{0.67}$	[45]
Evaporator	$\log C_{0,Eva}=\left[K_{1,Eva}+K_{2,Eva}\left(\log A_{Eva}\right)+K_{3,Eva}{\left(\log A_{Eva}\right)}^{2}\right]$ $\log F_{P,Eva}=\left[C_{1,Eva}+C_{2,Eva}\left(\log P_{Eva}\right)+C_{3,Eva}{\left(\log P_{Eva}\right)}^{2}\right]$ $Z_{Eva}=C_{0,Eva}\times \left[B_{1,Eva}+\left(B_{2,Eva}\times F_{M,Eva}\times F_{P,Eva}\right)\right]$	[37]

The LCOE (levelized cost of energy) is an important economic indicator. Low-cost electricity generation can be lucrative for investors in a specific period, which relatively low LCOE value can convince the investors to finance an energy system [46]. The LCOE can be calculated as [47]:

(21) $$LCOE=\frac{C_{TCI}+\sum_{t=1}^{n}\left(\frac{C_{TPC}}{{\left(1+i\right)}^{t}}\right)}{\sum_{t=1}^{n}\left(\frac{E_{t}}{{\left(1+i\right)}^{t}}\right)}$$

where $E_{t}$ is the generated electrical energy in year t $\left(\mathrm{kWh}\right)$. The pay-back period (PBP) is the required time for an energy system to provide a net zero return on investment by selling the produced energy. This parameter provides investors with an understanding of the risk of their investment, and can be calculated as follows [47]:

(22) $$PBP=\frac{C_{TDC}}{Cash\;flow}$$

The pay-back period is calculated according to corporate tax rates and average electricity prices for Iran’s industry.

## 4. Exergoenvironmental Impact Modeling

Analyzing the geothermal power plants in terms of environmental impacts besides energy and economic assessments is important. The exergoenvironmental impact factor is an indicator that represents the environmental impact of the power cycle due to irreversibilities during the power plant operation that can be calculated as follows [48,49]:

(23) $$f_{ei}=\frac{\dot{E}xD_{Tot}}{\dot{E}x_{i}}$$

The exergoenvironmental impact index is an indicator that represents how environmentally damaging a system is. It can be written as [48,49]:

(24) $$\theta_{ei}=\frac{100\times f_{ei}}{\eta_{ex}}$$

This indicator shows if an energy system damages the environment or not. An environmentally friendly system should have lower exergoenvironmental impact index values. Exergoenvironmental impact improvement is another index that indicates the relation between the environment and the energy system. From an environmental point of view, a desirable energy system has a higher value of exergoenvironmental impact improvement. This factor can be expressed [48,49]:

(25) $$\theta_{eii}=\frac{1}{\theta_{ei}}$$

Another important indicator is the exergetic stability factor, which can be written as follows [48,49]:

(26) $$f_{es}=\frac{\dot{E}x_{out}}{\dot{E}x_{out}+\dot{E}x_{D,Tot}+\dot{E}x_{uu}}$$

Here, $\dot{E}x_{uu}$ is fuel exergy consumption rate by the energy system. In an ideal energy system, this value is equal to 1. The exergetic sustainability index is another factor that provides useful insights. It can be written as follows [48,49]:

(27) $$\theta_{est}=f_{es}\times \theta_{eii}$$

For a favorable energy system, this index should be as high as possible.

## 5. Multi-Objective Optimization

**Figure 4:**
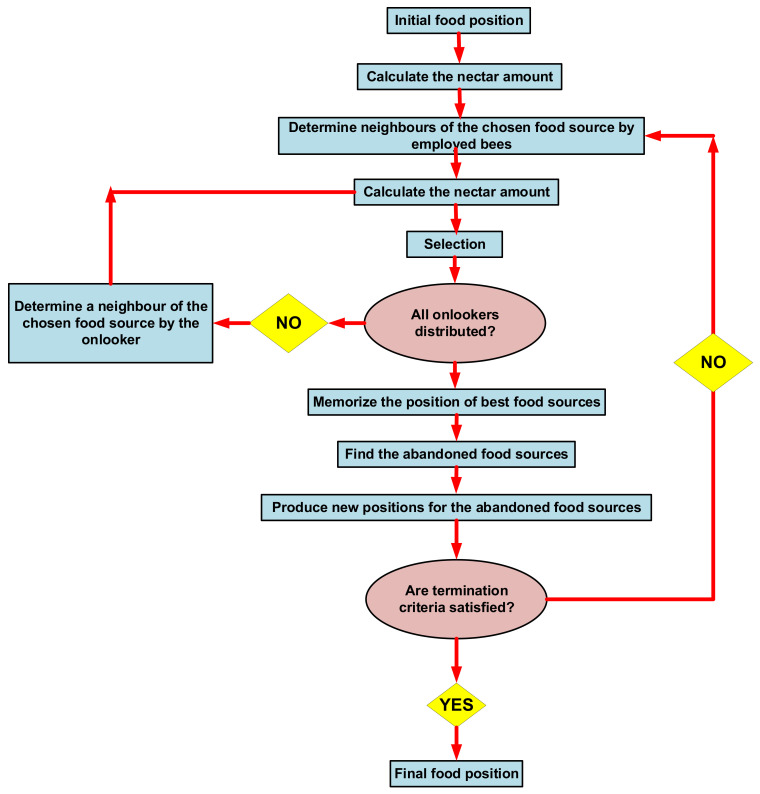
Flowchart of the artificial bee colony algorithm [51].

All cycles considered here are optimized from energy and economic viewpoints with the artificial bee colony algorithm. This algorithm has been selected for optimizing the cycles as it has some advantages, such as the ability to handle the objective cost, flexibility, the ability to explore local solutions, broad applicability, robustness, and complex functions. Two objective functions are considered: exergy efficiency and product electricity cost. The final goal of optimization is minimizing electricity cost, and maximizing efficiency. Product electricity cost could be considered as a thermo-economic objective function. However, based on the optimizing process, the optimum point for electricity cost was not corresponding with exergy efficiency optimum values. As exergy efficiency depends on destruction and losses, however, electricity cost is affected by PEC and other economic element variations besides the thermodynamic performance of the cycle. The advantage of this multi-objective optimization, compared with single objective optimization, is better performance in both thermodynamic and economic aspects. Considering just product electricity cost for a single objective optimization helped to minimize it, but harms exergy efficiency. The decision variables and their constraints in the optimization process are listed in Table 4. The artificial bee colony algorithm, proposed by Karaboga [50], simulates the foraging behavior of a bee colony. This algorithm is able to optimize numerical problems [51]. In the artificial bee colony algorithm, the food source location shows a possible solution to the optimization problem. The amount of nectar from a food source corresponds to the associated solution’s quality (fitness). The number of the population solution is the number of the employed bees [52]. A flowchart of the artificial bee colony algorithm is presented in Figure 4.

## 6. Results and Discussion

**Figure 5:**
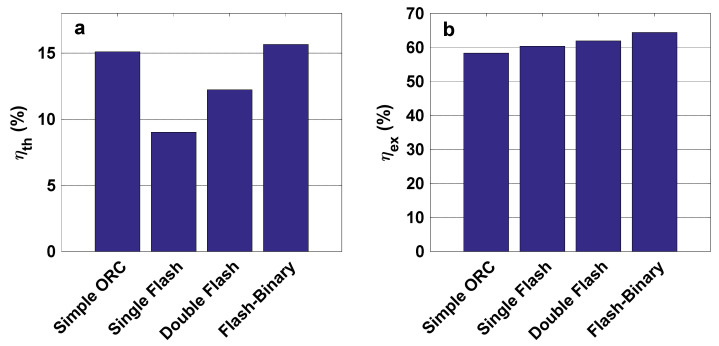
(**a**) Thermal and (**b**) exergy efficiencies of all considered configurations.

**Figure 6:**
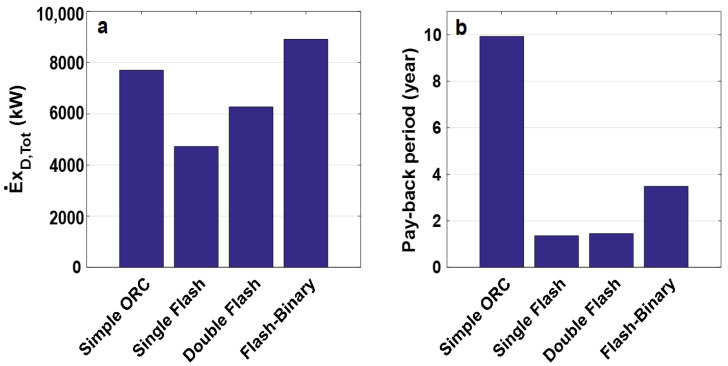
(**a**) Exergy destruction rate and (**b**) pay-back period of all considered configurations.

Figure 5a,b show the thermal and exergy efficiencies of all evaluated cycles. According to the obtained results, the flash-binary cycle has the highest thermal and exergy efficiencies, mainly related to decreasing the heat waste of the flash system, and using this energy in electricity production as a coupled ORC system. Among these cycles, the single flash and simple ORC cycles have the lowest thermal and exergy efficiencies, respectively. However, a simple ORC cycle has the highest thermal efficiency after flash-binary. The maximum thermal efficiencies do not correspond to the highest exergy efficiencies, as the exergy efficiency depends on exergy destruction and exergy loss, fuel, and product. The exergy destruction rate and pay-back period of all configurations are displayed in Figure 6a,b. It can be observed that the flash-binary cycle has the highest exergy destruction rate, as this cycle includes more equipment than others. The lengthiest pay-back period is attributable to the simple ORC cycle, primarily because of the high value of evaporator cost based on its capacity and heat.

**Figure 7:**
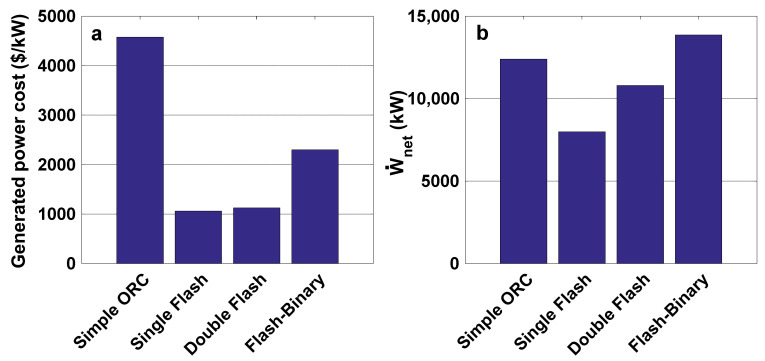
(**a**) Generated power cost and (**b**) net work rate of all considered configurations.

Furthermore, the single flash cycle has the shortest pay-back period. Figure 7a,b illustrate the generated power cost and net work rate for all assessed cycles. It can be seen that the highest generated power cost is related to the simple ORC, and the lowest to the single flash cycle. The power cost is slightly higher for the double flash than the single flash cycle. Moreover, the flash-binary cycle generates a higher net work rate than others.

**Figure 8:**
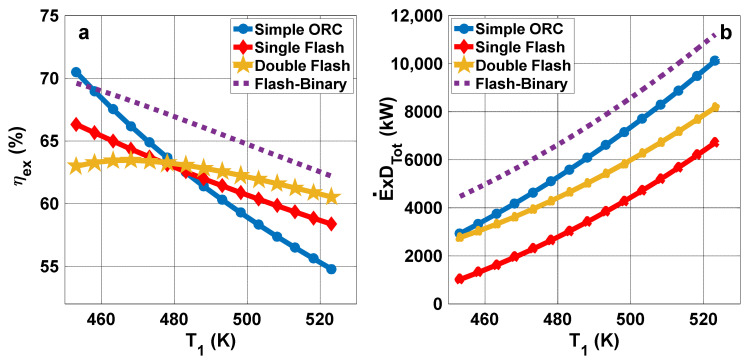
(**a**) Exergy efficiency and (**b**) exergy destruction rate variations with the temperature of the production well.

Figure 8a presents the variations of exergy efficiency with production well temperature. It is clear that increasing the well temperature leads to a decrease in the exergy efficiency of all cycles. However, in the case of the double flash cycle, the exergy efficiency increases as the temperature rises to 470 K, and then decreases as the production well temperature rises further. The exergy destruction for all cases rises with well temperature, as shown in Figure 8b.

**Figure 9:**
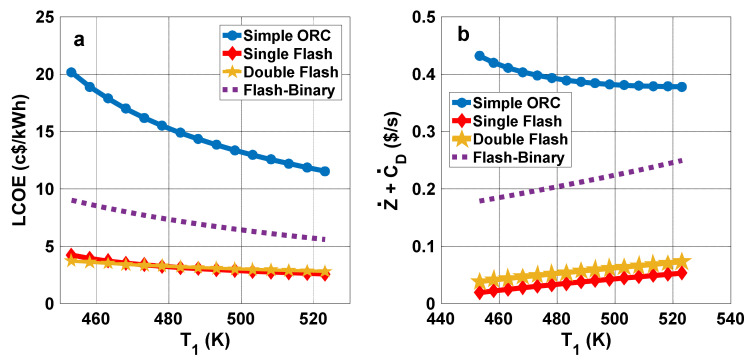
(**a**) LCOE and (**b**) total cost rate variations with production well temperature.

The impact of the production well temperature on LCOE for the cycles is shown in Figure 9a. It can be seen that LCOE decreases with rising production well temperature in all cases. This decline is steeper for the simple ORC relative to the other cycles. According to Figure 9b, the total cost rate for the simple ORC diminishes with rising production well temperature, but it rises in other cases.

**Figure 10:**
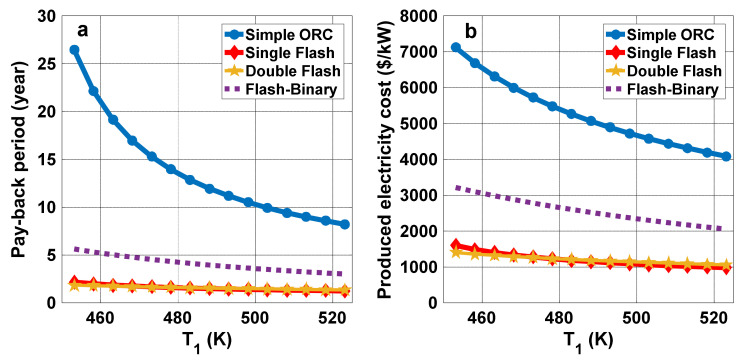
(**a**) Pay-back period and (**b**) produced electricity cost variations with production well temperature.

Figure 10a shows the variation of the pay-back period with production well temperature. The pay-back period is observed to decline with rising production well temperature for all considered cases. The decrease is more pronounced for the simple ORC. The product electricity cost variation with production well temperature is illustrated in Figure 10b. In all cases, the electricity cost decreases as production well temperature rises, and that trend is more significant for the simple ORC.

**Figure 11:**
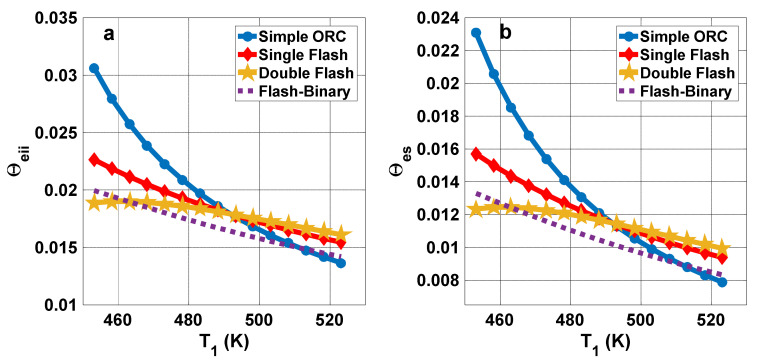
(**a**) Exergoenvironmental impact improvement factor and (**b**) exergetic sustainability index variations with production well temperature.

Figure 11a displays the effect of production well temperature on the exergoenvironmental impact improvement factor. It is seen for all cycles that the exergoenvironmental impact improvement factor decreases as production well temperature rises. This trend is consistent with the exergy destruction variations trend, since the irreversibilities increase with rising production well temperature. However, in the double flash cycle, exergoenvironmental impact improvement factor first rises with production well temperature to 470 K, and then declines as the temperature rises further. These trends are similar for the exergetic sustainability index variations with production well temperature, as shown in Figure 11b. That is, increasing the production well temperature is seen to reduce the system’s exergetic sustainability performance.

**Figure 12:**
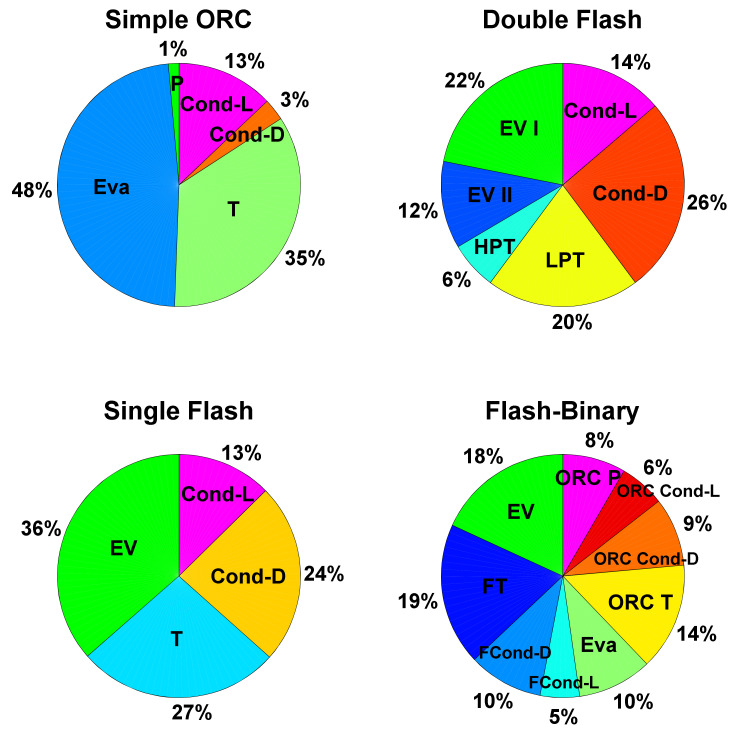
Exergy destruction ratio of components for each system configuration.

**Figure 13:**
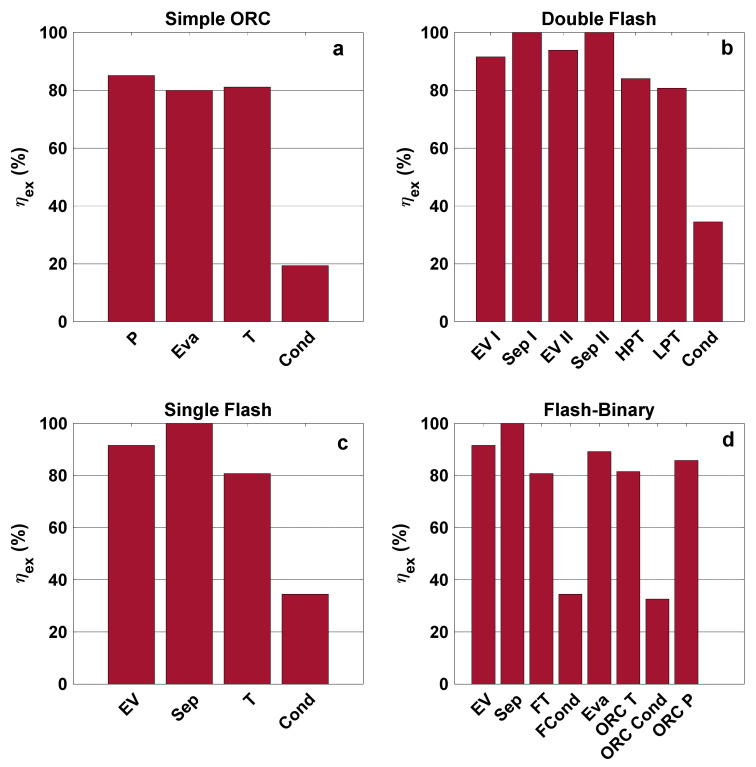
Exergy efficiencies of components for each system configuration.

Figure 12 presents the exergy destruction ratio for all assessed configurations. It can be seen that, for all cycles, the turbine has the highest destruction ratio, except in simple ORC, where the evaporator has the highest destruction ratio. In the single flash cycle, the evaporator exergy destruction ratio has the highest exergy destruction after turbine with insignificant difference. For the simple ORC, however, the evaporator has the highest exergy destruction ratio. The exergy efficiencies of the components in all configurations are displayed in Figure 13. The product and fuel costs of each component of all configurations are presented in Table 5.

**Figure 14:**
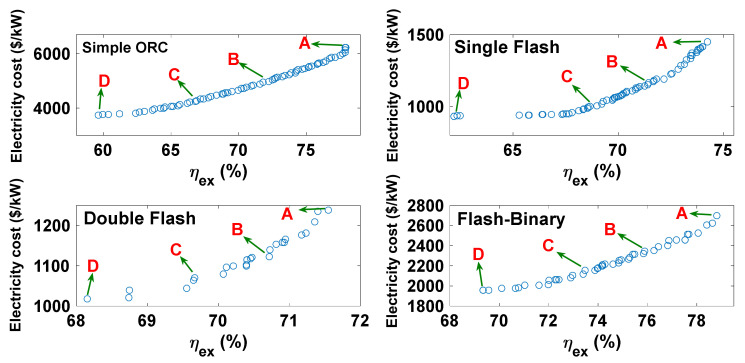
Pareto curves for all cycles.

Figure 14 displays the Pareto curves derived from the optimization routines for all configurations. Four points (A–D) are selected to compare the optimum point results. The values of the objective functions at these selected points are listed in Table 6. According to the optimum results and the base case value, point C is selected as the optimum point for each cycle. Point C is chosen since the optimization goal is normalizing both objective functions simultaneously. Points A and B in all cycles have higher electricity costs than the base case, so they are not considered optimal solutions. The values of the objective functions at the selected optimum point (point C) and the base case for all evaluated configurations are listed in Table 7. The base case is the design point based on the parameters presented in Table 1. The values of the design variables at the optimum and base case states are presented in Table 8. The values of the main parameters at the optimum and base case states are compared in Table 9. It is seen that, at optimum state, the exergy efficiencies of the simple organic Rankine, single flash, double flash, and flash-binary cycles are 14.7%, 14.4%, 12.6%, and 14.1% higher than the corresponding efficiencies for the base state, respectively. Moreover, the electricity costs of the simple organic Rankine, single flash, double flash, and flash-binary plants are 6.9%, 5.2%, 4.8%, and 6.3% lower than the corresponding values for the base case, respectively.

The optimization results show that the LCOEs of the simple organic Rankine, single flash, double flash, and flash-binary cycles at the optimum point are 6.2%, 3.2%, 1%, and 1.4% lower than the base case, respectively. Moreover, the pay-back periods for the simple organic Rankine, single flash, double flash, and flash-binary cycles at the optimum point are 10.6%, 1.5% 1.4%, and 0.6% lower than the base state, respectively.

## 7. Conclusions

In the present study, energy, exergy, and exergoeconomic analyses of various geothermal power plant configurations are performed. Modeling is carried out in MATLAB, and mass and energy balance equations are employed. The considered geothermal power cycle configurations are simple organic Rankine, single flash, double flash, and flash-binary. Multi-objective optimization of these configurations is carried out with the artificial bee colony algorithm. A sensitivity analysis is performed of the effect of production well temperature variations on the energy, exergy, and economic parameters of the systems. The results show that the flash-binary cycle has the highest thermal and exergy efficiencies among these cycles, at 15.6% and 64.3%, respectively. The highest generated power cost and pay-back period are related to the simple ORC. It is seen that an increase in the production well temperature raises the exergy destruction rate for all configurations. However, the electricity costs and pay-back periods decrease. Further, for all cases, the exergoenvironmental impact improvement factor decreases with production well temperature. However, in the double flash cycle, the exergoenvironmental impact improvement factor rises temperature to 470 K, and declines thereafter.

The turbine is observed to have the highest destruction rate in all cycles, with the exception of the evaporator having the highest destruction rate for the simple ORC. Moreover, the lowest exergy efficiency for all cases is associated with the condenser. At optimum state, the exergy efficiencies of the simple organic Rankine, single flash, double flash, and flash-binary cycles are found to be 14.7%, 14.4%, 12.6%, and 14.1% higher than the corresponding base states, respectively. The optimization results show that the LCOE of the simple organic Rankine, single flash, double flash, and flash-binary cycles at the optimum point are 6.2%, 3.2%, 1.0%, and 1.4% lower than the base case, respectively. Moreover, the pay-back periods for the simple organic Rankine, single flash, double flash, and flash-binary cycles at the optimum point are 10.6%, 1.5% 1.4%, and 0.6% lower than the base state, respectively.

## Figures and Tables

**Table 4:** Optimization design variables and constraints for each configuration.

Cycle	Decision Variable	Lower Bound	Upper Bound
Simple ORC	$T_{1}\;\left(K\right)$	453	523
	$P_{4}\;\left(\mathrm{kPa}\right)$	2000	3300
	$\eta_{T}\;\left(\%\right)$	70	90
	$\eta_{P}\left(\%\right)$	70	90
	$T_{6}\;\left(K\right)$	303	313
Single flash	$T_{1}\;\left(K\right)$	453	523
	$P_{2}\;\left(\mathrm{kPa}\right)$	400	800
	$\eta_{T}\left(\%\right)$	70	90
Double flash	$T_{1}\;\left(K\right)$	453	523
	$P_{2}\;\left(\mathrm{kPa}\right)$	400	800
	$\eta_{HPT}\left(\%\right)$	70	90
	$\eta_{LPT}\left(\%\right)$	70	90
	$P_{9}\;\left(\mathrm{kPa}\right)$	50	300
Flash-binary	$T_{1}\;\left(K\right)$	453	523
	$P_{2}\;\left(\mathrm{kPa}\right)$	400	800
	$\eta_{FT}\left(\%\right)$	70	90
	$\eta_{ORCT}\left(\%\right)$	70	90
	$\eta_{ORCP}\left(\%\right)$	70	90
	$T_{10}\;\left(K\right)$	303	313

**Table 5:** Product and fuel costs of each component for all configurations.

**Component**	**Simple ORC**		**Component**	**Single Flash**	
	${\dot{C}}_{p}\;\left(\$/s\right)$	${\dot{C}}_{f}\;\left(\$/s\right)$		${\dot{C}}_{p}\;\left(\$/s\right)$	${\dot{C}}_{f}\;\left(\$/s\right)$
P	0.0239	0.0212	EV	0	0.0079
Eva	0.2505	0.0078	Sep	0.0081	0.0080
T	0.3360	0.2849	T	0.0381	0.0036
Cond	0	0.0639	Cond	0	0.0005
**Component**	**Double flash**		**Component**	**Flash-binary**	
	${\dot{C}}_{p}\;\left(\$/s\right)$	${\dot{C}}_{f}\;\left(\$/s\right)$		${\dot{C}}_{p}\;\left(\$/s\right)$	${\dot{C}}_{f}\;\left(\$/s\right)$
EV I	0	0.0079	EV	0	0.0079
Sep I	0.0081	0.0080	Sep	0.0081	0.0080
EV II	0	0.0043	FT	0.0399	0.0041
Sep II	0.0044	0.0044	FCond	0	0.0006
HP T	0.0132	0.0009	Eva	0.0903	0.0034
LP T	0.0414	0.0045	ORC T	0.0316	0.0011
Cond	0	0.0008	ORC Cond	0	0.0662
			ORC P	0.0039	0.0023

**Table 6:** Values of objective functions at selected points on the Pareto curve for all cycles.

Cycle	Objective Function	A	B	C	D
Simple ORC	Exergy efficiency $\left(\%\right)$	77.9	72.3	66.9	59.6
	Electricity cost $\left(\$/\mathrm{kW}\right)$	6238	4982	4260	3746
Single flash	Exergy efficiency $\left(\%\right)$	74.2	71.4	69	62.2
	Electricity cost $\left(\$/\mathrm{kW}\right)$	1451	1165	1006	932
Double flash	Exergy efficiency $\left(\%\right)$	71.6	70.7	69.7	68.1
	Electricity cost $\left(\$/\mathrm{kW}\right)$	1239	1123	1070	1018
Flash-binary	Exergy efficiency $\left(\%\right)$	78.8	75.9	73.5	69.3
	Electricity cost $\left(\$/\mathrm{kW}\right)$	2699	2349	2153	1959

**Table 7:** Values of objective functions at the selected optimum point.

Cycle	$\eta_{ex}\;\left(\%\right)$			Electricity Cost $\left(\$/\mathrm{kW}\right)$		
	Optimum	Base Case	Difference	Optimum	Base Case	Difference
Simple ORC	66.9	58.3	+14.7%	4260	4575.2	−6.9%
Single flash	69	60.3	+14.4%	1006	1061.1	−5.2%
Double flash	69.7	61.9	+12.6%	1070	1124.2	−4.8%
Flash-binary	73.5	64.4	+14.1%	2153	2298.7	−6.3%

**Table 8:** Values of design variables at the optimum and base case for all cycles.

Cycle	Design Variable	Optimum Value	Base Case Value
Simple ORC	$T_{1}\;\left(K\right)$	497.7	503
	$P_{4}\;\left(\mathrm{kPa}\right)$	2890.2	3000
	$\eta_{T}\;\left(\%\right)$	88	85
	$\eta_{P}\;\left(\%\right)$	89.8	85
	$T_{6}\;\left(K\right)$	303.1	303
Single flash	$T_{1}\;\left(K\right)$	497	503
	$P_{2}\;\left(\mathrm{kPa}\right)$	724.2	600
	$\eta_{T}\;\left(\%\right)$	89.1	85
Double flash	$T_{1}\;\left(K\right)$	490	503
	$P_{2}\;\left(\mathrm{kPa}\right)$	703.6	600
	$\eta_{HPT}\;\left(\%\right)$	81.8	85
	$\eta_{LPT}\;\left(\%\right)$	89.6	85
	$P_{9}\;\left(\mathrm{kPa}\right)$	245.6	200
Flash-binary	$T_{1}\;\left(K\right)$	490.2	503
	$P_{2}\;\left(\mathrm{kPa}\right)$	563	600
	$\eta_{FT}\;\left(\%\right)$	88.9	85
	$\eta_{ORCT}\;\left(\%\right)$	88	85
	$\eta_{ORCP}\;\left(\%\right)$	76.5	85
	$T_{10}\;\left(K\right)$	305.4	313

**Table 9:** Main parameters for all configurations at the optimum and base case states.

**Parameter**	**Simple ORC**			**Single Flash**		
	**Base**	**Optimum**	**Difference**	**Base**	**Optimum**	**Difference**
${\dot{W}}_{net}\left(\mathrm{kW}\right)$	12,381	13,241.2	+6.9%	7990	7580.1	−5.1%
${\dot{Z}}_{Tot}+{\dot{C}}_{D}\;\left(\$/s\right)$	0.381	0.355	−6.8%	0.044	0.041	−6.8%
$LCOE\;\left(c\$/\mathrm{kW}\right)$	12.9	12.1	−6.2%	2.8	2.71	−3.2%
$PBP\;\left(\mathrm{year}\right)$	9.9	8.85	−10.6%	1.36	1.34	−1.5%
$PEC_{Tot}\;\left(\$\right)$	4.2 × 10^6^	4.1 × 10^6^	−2.4%	5.9 × 10^6^	5.6 × 10^6^	−5%
**Parameter**	**Double Flash**			**Flash-Binary**		
	**Base**	**Optimum**	**Difference**	**Base**	**Optimum**	**Difference**
${\dot{W}}_{net}\left(\mathrm{kW}\right)$	10,827	10,068.8	−7%	13,856	14,340.7	+3.5%
${\dot{Z}}_{Tot}+{\dot{C}}_{D}\;\left(\$/s\right)$	0.064	0.058	−9.4%	0.227	0.222	−2.2%
$LCOE\;\left(c\$/\mathrm{kW}\right)$	2.98	2.95	−1%	6.3	6.21	−1.4%
$PBP\;\left(\mathrm{year}\right)$	1.45	1.43	−1.4%	3.48	3.46	−0.6%
$PEC_{Tot}\;\left(\$\right)$	8.5 × 10^6^	7.9 × 10^6^	−7%	2.3 × 10^7^	2.1 × 10^7^	−8.7%

## Data Availability

Not applicable.

## Nomenclature

c	Specific exergy cost $\left(\$/\mathrm{kJ}\right)$
$\dot{C}$	Cost rate associated with exergy transfer $\left(\$/s\right)$
CEPCI	Chemical Engineering Plant Cost Index
CRF	Capital Recovery Factor
ex	Specific exergy $\left(\mathrm{kJ}/\mathrm{kg}\right)$
$\dot{Ex}$	Exergy rate $\left(\mathrm{kW}\right)$
f	Exergoeconomic factor
$f_{ei}$	Exergoenvironmental impact factor
$f_{es}$	Exergetic stability factor
h	Specific enthalpy $\left(\mathrm{kJ}/\mathrm{kg}\right)$
i	Rate of interest
LCOE	Levelized Cost of Electricity $\left(\$/\mathrm{kWh}\right)$
$\dot{m}$	Mass flow rate $\left(\mathrm{kg}/s\right)$
N	Annual plant operation hours
n	Power plant lifetime
ORC	Organic Rankine cycle
P	Pressure $\left(\mathrm{kPa}\right)$
PBP	Pay-back period $\left(\mathrm{year}\right)$
PEC	Purchased Equipment Costs ($)
$\dot{Q}$	Heat transfer rate $\left(\mathrm{kW}\right)$
r	Relative cost difference
s	Specific entropy $\left(\mathrm{kJ}/\mathrm{kgK}\right)$
T	Temperature $\left({}^{{}^{\circ}}C\right)$
$\dot{W}$	Power $\left(\mathrm{kW}\right)$
x	Quality
y	Exergy destruction ratio
z	Well depth
Z	Capital cost of components $\left(\$\right)$
$\dot{Z}$	Capital cost rate of components $\left(\$/s\right)$
**Greek Symbols**	
η	Efficiency $\left(\%\right)$
φ	Maintenance factor
$\theta_{ei}$	Exergoenvironmental impact index
$\theta_{eii}$	Exergoenvironmental impact improvement factor
$\theta_{est}$	Exergetic sustainability index
**Subscripts**	
Cond	Condenser
D	Destruction
EV	Expansion valve
Sep	Separator
FT	Flash turbine
ORCT	ORC turbine
P	Pump
Eva	Evaporator
F	Fuel
P	Product
Ph	Physical
q	Specific heat rate
w	Specific work rate
Tot	Total
i	inlet
e	exit
0	Ambient
